# Decoding Cytoskeletal Mechanobiology with Tunable Microenvironments

**DOI:** 10.1021/acsbiomaterials.6c00339

**Published:** 2026-10-12

**Authors:** Rupesh Kandel, Amzadul Hoque Chowdhury, Jacqueline Robinson, Dana N. Reinemann

**Affiliations:** Department of Biomedical Engineering, University of Mississippi, Oxford, Mississippi 38677, USA; Department of Biomedical Engineering, University of Mississippi, Oxford, Mississippi 38677, USA; Department of Biomedical Engineering, University of Mississippi, Oxford, Mississippi 38677, USA; Department of Biomedical Engineering, University of Mississippi, Oxford, Mississippi 38677, USA; Department of Chemical Engineering, University of Mississippi, Oxford, Mississippi 38677, USA

**Keywords:** cytoskeleton, tunable surfaces, biointerfaces, mechanobiology

## Abstract

Mechanical cues from the cellular microenvironment are key regulators of cytoskeletal organization, force generation, and mechanotransduction, yet defining how specific mechanical inputs control distinct cytoskeletal processes remains an ongoing challenge. Mechanically tunable microenvironments have become essential tools for addressing this problem by enabling systematic variation of substrate stiffness, viscoelasticity, and interfacial mechanics under well-controlled conditions. In this review, we survey major classes of tunable platforms used to study cytoskeletal dynamics, including biologically derived matrices, synthetic hydrogels, micropatterned and nanotopographic substrates, and polymeric thin films, highlighting how each approach affords unique insight into cytoskeletal regulation. Beyond material composition, these platforms reveal key mechanistic principles: they allow separation of protrusion, contractility, and adhesion processes; uncover how mechanical coupling drives alignment and polarity; and identify stiffness thresholds that govern force transmission. Control over interface mechanics also enables selective engagement of cytoskeletal modules that are otherwise inseparable on rigid substrates. We conclude by discussing current limitations and emerging opportunities for integrating tunable microenvironments with quantitative measurements of force generation and cytoskeletal organization, advancing a more predictive understanding of how cells sense, transmit, and respond to mechanical information.

## Graphical Abstract

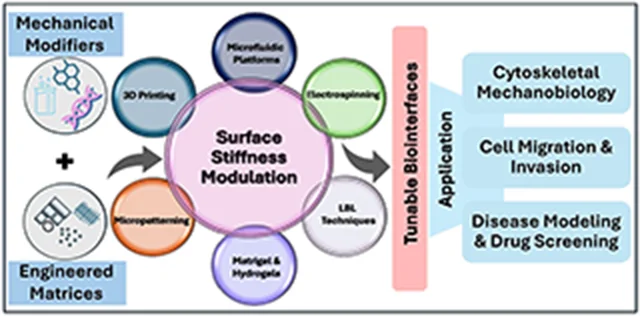

## 1. INTRODUCTION

Engineered biomaterials and tunable substrates have emerged as powerful tools to systematically probe how cells regulate cytoskeletal organization in response to mechanical forces. By modulating surface properties such as elasticity, nanotopography, and ligand density, researchers can precisely control the mechanical environment of cells and investigate biophysical mechanisms underlying cytoskeletal remodeling.^1,2^ Importantly, these platforms are not only used to mimic physiological environments but also to isolate specific mechanical parameters and link them to distinct cytoskeletal responses, enabling identification of mechanisms that are difficult to resolve on conventional rigid substrates.^3^ Through mechanosensing and mechanotransduction pathways, cells convert mechanical stimuli into biochemical signals, regulating fundamental physiological and pathological processes.^4,5^ For example, cells cultured on soft substrates exhibit more dynamic, less contractile actin structures, while those on stiffer surfaces develop stress fibers and exert higher traction forces.^6^ Similarly, nanoscale surface features influence focal adhesion maturation, intracellular force transmission, and migration efficiency.^7^

Despite significant progress, several questions remain regarding the molecular mechanisms that govern cytoskeletal adaptations to mechanical stimuli. How do individual cytoskeletal components coordinate their response to extracellular mechanical inputs? What signaling pathways mediate the conversion of mechanical forces into biochemical responses? A central challenge is therefore to systematically connect defined mechanical inputs, such as stiffness, viscoelasticity, and interfacial architecture, to specific cytoskeletal outputs, including adhesion dynamics, contractility, and force propagation. Combining advanced imaging techniques, high-resolution force measurements, and synthetic biomaterials will provide deeper insights into how surface mechanical properties influence cytoskeletal dynamics and, ultimately, cell function.^8,9^

One emerging challenge is the development of surface modification techniques that closely mimic the extracellular matrix (ECM). Classical methods such as Langmuir-Blodgett films,^10^ self-assembled multilayers,^11^ polymer grafting,^12^ and chemical vapor deposition^13^ have been widely used for immobilizing biomacromolecules on surfaces. Biomimetic ECM-like surfaces should capture both biochemical composition and dynamic mechanical properties that regulate cytoskeletal behavior.^2^ Changes in the ECM environment, such as modifications in topography, mechanical stiffness, and biocompatibility, can significantly influence cytoskeletal behavior.^2^ Despite its advantages, relatively limited work has focused on cytoskeletal dynamics within tunable microenvironments using reconstituted or minimal biomimetic platforms, highlighting an important gap in the field.^5^

This review examines how mechanically tunable surfaces have been used to investigate cytoskeletal regulation in cell-based systems, with an emphasis on how these platforms attempt to bridge the gap between *in vitro* studies and the mechanical complexity of *in vivo* environments. Although many cytoskeletal processes are still studied on mechanically static, rigid surfaces that poorly recapitulate physiological mechanics, such simplified *in vitro* systems provide experimental control for isolating specific molecular and biophysical mechanisms. Here, along with fabrication strategies, we emphasize how specific material parameters, such as stiffness, viscoelasticity, and interfacial structure, have been used to uncover mechanistic insights into cytoskeletal organization and force regulation. We further highlight how different classes of platforms provide access to distinct mechanical regimes, enabling selective interrogation of cytoskeletal processes such as adhesion, contractility, and protrusion. By enabling systematic variation of mechanical and structural cues, these systems have expanded the range of mechanobiological questions that can be addressed in cellular contexts. Finally, we discuss the limitations of current substrate-based approaches and outline opportunities to bridge cell-based mechanobiology with more reductionist, reconstituted cytoskeletal systems, in which mechanical regulation can be examined with greater control over molecular composition.

## 2. APPROACHES TO MODIFYING SURFACE STIFFNESS

### 2.1. Matrigel and Hydrogels

Matrigel, a commercially available basement membrane extract derived from Engelbreth-Holm-Swarm (EHS) mouse sarcoma, is widely used in biomedical research to mimic the extracellular matrix (ECM)^14^ and study cell behavior.^15^ Matrigel creates a tunable microenvironment on assay surfaces, such as well plates, flasks, scaffolds, and glass coverslips, which support cell adhesion, proliferation, differentiation, and cytoskeletal organization, provided the surface allows stable Matrigel coating.^16^ Its soft, gel-like mechanical properties, with an elastic modulus typically ranging from 0.1 to 1 kPa, make it particularly suitable for studying mechanosensitive cellular processes, including cytoskeletal dynamics.^17^ In this context, Matrigel has been widely used to reveal how cells adapt their cytoskeletal architecture in response to compliant, three-dimensional environments that more closely resemble the mechanics of native tissue. Sun et al. reported the 3D Matrigel culture system effectively promoted neurite outgrowth of spiral ganglion neurons (SGNs), preserving the delicate architecture of SGN explants without inducing abnormal neurite growth patterns.^18^ This outcome is attributed to the ability of Matrigel to closely mimic the native three-dimensional organization of SGNs under physiological conditions.^18^ Poincloux et al. demonstrated that invasive breast cancer cells navigating 3D Matrigel microenvironment critically depend on actomyosin contractility for successful migration, highlighting the coordinated role of cytoskeletal dynamics and matrix adhesion in tumor cell development (Figure 1A).^19^ These observations demonstrate that in soft, 3D matrices, cytoskeletal force generation is not solely dictated by substrate stiffness but emerges from a balance between actomyosin contractility and matrix resistance, providing insight into how cells regulate migration under physiologically relevant mechanical constraints. Together, these studies position Matrigel as an illustrative example of how soft, biologically complex matrices shape cytoskeletal dynamics while also highlighting the limitations imposed by restricted control over mechanical and biochemical parameters.

In addition to Matrigel, other biocompatible materials such as hydrogels have been developed to provide more controlled and tunable microenvironments for studying cytoskeletal dynamics.^20^ Hydrogels constitute a foundational class of mechanically tunable substrates for cytoskeletal studies because they allow bulk matrix compliance to be varied over physiologically relevant ranges, enabling systematic investigation of how cytoskeletal organization, contractility, and force transmission respond to global mechanical cues.^15^ This capability has enabled the identification of stiffness-dependent transitions in cytoskeletal behavior, where cells exhibit distinct regimes of spreading, adhesion maturation, and contractility across defined mechanical thresholds.^15^ PEG hydrogels are valued for their tunable mechanical properties and biocompatibility, making them ideal for studying cellular responses to stiffness gradients.^21^ By adjusting the crosslinking density poly(ethylene glycol) diacrylate (PEG-DA molecular weight or photoinitiator concentration), their elastic modulus can be tuned across a physiologically relevant range of ~0.1 to 100 kPa,^22^ encompassing soft tissues (~0.1–1 kPa) to stiffer matrices (~30–100 kPa). Alexander et al. reported that the PEG-based hydrogels can be chemically modified with cell-adhesive peptides (e.g., arginyl-glycyl-aspartic (RGD) sequences) and crosslinked to achieve precise control over stiffness, enabling detailed investigations into cytoskeletal responses to mechanical cues within 3D microenvironments.^23^ Such systems have been particularly useful for decoupling mechanical stiffness from biochemical signaling, allowing direct assessment of how cytoskeletal organization and force generation are regulated by matrix elasticity, independent of ligand density.^23^ Figure 1B shows the organization of cardiomyocytes on carbon nanotubes incorporated into a gelatin derivative (CNT–GelMA) surfaces examined under static culture conditions.^20^ The high electrical conductivity and mechanical tunability of CNTs lead to extensive nanofibrous networks in the porous gelatin framework, which improves the mechanical strength of the scaffold. The myocardial structures exhibited high mechanical integrity and advanced electrophysiological functions on 50 μm-thick CNT-GelMA compared to pristine GelMA hydrogels.^20^

To achieve fine control over mechanical parameters, a range of synthetic and semi-synthetic hydrogels has been developed that enable independent tuning of stiffness, crosslinking density, and biochemical functionalization.^22^ Naturally derived hydrogels, such as alginate hydrogels, offer tunable stiffness (~1– 50 kPa) through variations with calcium (Ca^2+^) concentrations, where higher Ca^2+^ (50–100 mM) yields stiffer networks during crosslinking.^24^ An increase in the effective Young’s modulus was observed in hydrogels containing hyaluronic acid (HA)^25^ compared to collagen-only (Col) gels, where HA’s influence on the modulus was dependent on the cell type and the coculture ratio.^26^

Furthermore, polyacrylamide (PA) gels are extensively utilized as synthetic substrates for creating tunable mechanical environments in cellular studies.^27^ By modulating the crosslinking density through variations in the ratio of acrylamide to bis-acrylamide, the elastic modulus of these gels can be precisely controlled, spanning a wide range from a few hundred Pascal (Pa) to several kilopascal (kPa).^27^ This flexibility allows PA gels to mimic the mechanical properties of various biological tissues, from soft brain-like matrices (~0.1 kPa) to rigid bone-like substrates (~100 kPa).^27^ Zhang et al. evaluated polyacrylamide gels that allow for precise control over substrate stiffness by varying the crosslinking density, while polydimethylsiloxane (PDMS) provides a wide range of elastic moduli suitable for studying cellular responses to mechanical cues.^28^ PDMS is a silicone-based elastomer that allows for the fabrication of substrates with adjustable mechanical properties by varying the base-to-crosslinker ratio. Its elastic modulus can range from ~1 kPa to several MPa, making it suitable for studying a wide range of mechanical cues.^29^ PDMS is often used in microfabrication and cell culture studies to investigate how surface mechanics affect cytoskeletal remodeling.^30^ Yeh et al. developed a two-step crosslinking strategy to increase the compressive modulus of PDMS substrates up to tenfold.^30^ This mechanical tuning led to increased cell spreading from ~1300 to 1900 μm^2^ in cardiac fibroblasts and from ~2700 to 4600 μm^2^ in mesenchymal stem cells as well as enhanced myofibroblast activation (from ~2% to 20%) with increasing stiffness.^30^ These findings highlight the cellular sensitivity to PDMS mechanics and demonstrate a photoresponsive, mechanically dynamic platform for modeling ECM behavior.^30^

Granular hydrogels, composed of densely packed microscale hydrogel building blocks, have emerged as an adaptable biomaterial platform, with formulations that exhibit solid-like behavior at low strain and reversible shear-thinning at high strain enabling tunable mechanics for advanced applications such as 3D bioprinting.^31^ Emiroglu et al. designed noncolloidal granular hydrogels by systematically linking microgel properties, specifically size and elasticity, to bulk rheological behavior.^31^ Engineered protein-based materials, including elastin-like polypeptides (ELPs), have further expanded the tunability of hydrogel and microgel systems through precise control of sequence, phase behavior, and mechanical properties.^32,33^

In addition to tuning the viscoelastic properties of engineered matrices, photochemical strategies have been used within cell-laden hydrogels.^34^ Photopatterned hydrogels are 3D crosslinked polymer networks that can be precisely structured using light (UV or visible) to generate defined geometries and localized biochemical or mechanical cues at the microscale.^35^ These systems allow orthogonal control over stiffness, porosity, and ligand presentation, making them useful platforms for studying cytoskeletal regulation and cell–matrix interactions in engineered microenvironments.^36^ Common approaches include photomask-based lithography, multiphoton laser scanning lithography, and light-triggered photocleavage or photo crosslinking chemistries, which enable highly resolved patterning in both 2D and 3D.^37^ In addition, photolithographic patterning has been widely used to define adhesive islands and stiffness gradients that regulate cell shape and traction force.^38^ Multiphoton-based patterning extends this control into fully 3D hydrogel matrices with submicron resolution.^39^

Microfluidic setups provide precise control over substrate stiffness by tuning hydrogel crosslinking density, polymer concentration, and network architecture directly within microscale channels.^40^ This system enables controlling the formation of tunable surfaces, allowing cells to experience spatially varying mechanical cues.^41^ Such control has been instrumental in demonstrating stiffness-dependent changes in cell spreading, focal adhesion assembly, and cytoskeletal tension, as well as directed migration along stiffness gradients.^42^ Flow-controlled delivery of crosslinkers or ions within microchannels further allows localized and reversible stiffness changes.^43^

Collectively, these platforms have established key principles of cytoskeletal mechanosensing under bulk mechanical constraints, particularly by revealing how global matrix stiffness regulates contractility, adhesion dynamics, and force transmission. While these approaches provide control over bulk mechanical properties, complementary platforms have been developed to investigate how cells respond to mechanical cues presented directly at the cell–material interface.^27^

### 2.2. Micropatterning, 3D Printing, and Electrospinning

#### 2.2.1. Micropatterning.

Micropatterned substrates with nanotopographical features can influence cell shape and cytoskeletal organization.^44^ Chen et al. developed a micropatterning approach that integrates microcontact printing with a dynamic attachment strategy to generate multicolor substrates containing three distinct functional regions: adhesive (microcontact-printed fibronectin), non-adhesive, and dynamically adhesive (biotinylated fibronectin captured via NeutrAvidin).^45^ Ghibaudo et al. demonstrated that 3D flexible microenvironments can regulate cell spreading using hexagonal arrays of fibronectin-coated micropillars fabricated via microfabrication techniques.^46^ This platform enabled precise spatial control over endothelial cell interactions, revealing that cells spread, formed adhesions, and exhibited motility to comparable extents on captured biotinylated fibronectin and on directly printed fibronectin. The flexible PDMS 3D micropillar arrays (Figure 2) have been utilized as engineered microenvironments to quantify the mechanical forces generated by non-muscle myosin II in association with the actin cytoskeleton.^46^ Forces were discretely transmitted along the pillars through focal adhesion sites, averaging ~5 nN per μm^2^ of adhesion area and producing total forces of ~250 nN consistent with the overall pillar surface (~300 μm^2^) engaged by cells over time.^46^

#### 2.2.2. 3D Printing.

3D printing, also known as additive manufacturing (AM) or rapid prototyping (RP), enables the fabrication of structures with precisely defined geometries in a layer-by-layer manner and thus constructs tunable microenvironments.^35^ Advanced 3D printing includes extrusion-based printing, stereolithography, and two-photon polymerization, which allows changing materials’ stiffness for multicellular constructs, which have been shown to direct cytoskeleton alignment, polymerization, and force generation in a highly controlled manner.^47^ Hydrogels comprising polymers (e.g., alginate and polyvinyl alcohol [PVA]) are the most commonly used bioinks for cell-laden 3D printing, as they provide a highly hydrated environment that supports cell viability and function.^15^ These biomaterials create mechanical support to encapsulated cells, as well as adhesion sites that facilitate cell attachment and migration.^48^ In addition, they present bioactive biochemical cues that regulate and guide complex cellular functions.^35^ Notably, the incorporation of 3D printing with Förster resonance energy transfer (FRET)-based tension sensors, optical tweezers, and live-cell fluorescence imaging has enabled direct measurement of cell-generated forces and cytoskeletal dynamics within defined 3D environments.^49^

#### 2.2.3. Electrospinning.

Electrospinning enables the fabrication of nanoscale to microscale fibrous platforms that closely resemble the ECM, creating a tunable environment.^50^ These platforms are created by using bioactive materials such as synthetic and natural polymers, typically polycaprolactone (PCL), polyurethane (PU), polyacrylonitrile, poly(L-lactic acid) (PLLA), chitosan, gelatin, cellulose, and silk.^51^ These techniques allow nanolevel surface modification to control surface hydrophilicity, oxide layer thickness, or functional group distribution to form ECM-like nanostructures that mimic biological environments and influence cell behavior, signal transduction, and nutrient transport.^52^ Zhu et al. synthesized biodegradable poly(ether carbonate urethane) urea (PECUU) with tunable Young’s moduli (2.5–13.4 MPa) by adjusting the stiffness of PECUU electrospun scaffolds to control the cellular microenvironment and modulate gene expression in annulus fibrosus-derived stem cells.^53^

### 2.3. Layer-by-Layer (LbL) Techniques

Layer-by-layer (LbL) assembled polymeric thin films are versatile composite biomaterials formed through the sequential deposition of molecular components onto a substrate.^54^ In mechanobiology, LbL assembly enables precise tuning of interfacial mechanical properties, allowing researchers to investigate how cytoskeletal processes respond specifically to surface-level cues rather than bulk material mechanics.^55^ Film assembly is typically driven by electrostatic interactions between oppositely charged molecules, although non-electrostatic interactions, including hydrogen bonding, covalent bonding, hydrophobic interactions, and coordination chemistry, have also been widely explored.^56^

The sequential and highly controllable nature of LbL assembly allows precise regulation of film thickness, composition, and mechanical properties at the nanoscale.^52^ These capabilities make LbL films particularly valuable for engineering mechanically tunable cellular interfaces while maintaining independent control over bulk substrate properties.^57^ As a result, LbL platforms provide a powerful framework for studying cell–material interactions, including how adhesion-mediated forces and cytoskeletal organization are regulated at the interface between cells and their surrounding environment.

Figure 3 summarizes deposition methods for LbL.^58^ After a specified number of cycles, a thin film with precisely controlled composition, thickness, and nanostructure is formed. Dip-coating is the most widely used method for LbL assembly, enabling uniform film deposition across a range of substrates.^59^ Alternative techniques, including spin-coating^60^ and spray-based approaches,^61^ have been developed to improve processing efficiency and scalability. While these methods differ in processing efficiency, due to adjustments in rotation speed and liquid viscosity, their relevance in mechanobiology lies in enabling the controlled fabrication of films with tunable interfacial mechanics and biochemical functionality.^61^

Park et al. developed brush LbL, which is based on the sequential brushing of polyelectrolyte solutions over a substrate.^62^ A wide range of synthetic and biological components, including polymers, proteins, and polysaccharides, can be incorporated into LbL films to tune mechanical properties and biochemical functionality.^63^ This compositional flexibility enables precise control over ligand presentation and interfacial mechanics, allowing systematic investigation of how cytoskeletal organization responds to combined mechanical and biochemical cues.^64^ The diversification of driving mechanisms and deposition methods has significantly broadened the scope of LbL assembly. Building blocks for LbL films now include synthetic polymers (charged or uncharged), such as polyethyleneimine (PEI),^63^ polystyrene sulfonate (PSS),^65^ polyallylamine hydrochloride (PAH),^66^ poly-L-lysine (PLL),^67^ and polyacrylic acid (PAA);^68^ biological polymers (e.g., collagen, gelatin,^69^ fibronectin,^70^ DNA, RNA);^71^ and polysaccharides (e.g., alginate, chitosan, hyaluronic acid,^72^ lignin,^73^ and heparin^74^) that exhibit intrinsic biocompatibility and tunable electrostatic interactions. Various biomolecules, including polysaccharides,^64^ enzymes,^75^ and peptides,^76^ have been incorporated into LbL films as either primary building blocks or functional surface modifications. These biomacromolecules exhibit diverse properties, such as bioactivity, bioavailability, and specificity, which confer multifunctionality to LbL films.

## 3. MECHANICAL PROPERTIES OF LBL FILMS

### 3.1. Stiffness of Materials

One of the defining advantages of LbL films is their tunable stiffness, with Young’s moduli spanning from tens of kPa to several MPa, providing a versatile platform to investigate how interfacial mechanical cues regulate cellular responses, including cytoskeletal organization, adhesion dynamics, and force transmission, in ways that are often distinct from bulk matrix effects.^77^ However, it is important to consider that most reported stiffness values of LbL films are measured in aqueous environments.^78^ Notably, the mechanical properties of LbL films vary significantly between their dry and hydrated states, with stiffness ranging from MPa to GPa in poly(allylamine hydrochloride)/poly (acrylic acid) (PAH/PAA) films.^79^ Adjusting polyelectrolyte interactions within LbL films generally yields stiffness values spanning from kPa to MPa.^80^ To attain even greater stiffness, extending into the GPa range, stiffer and more robust materials are integrated as structural components or fillers, as the mechanical properties of LbL films are inherently dependent on their composition.^81^ Table 1 lists the polycations and polyanions alongside the reported Young’s modulus value ranges.

In recent years, the development of nanocomposite LbL films has gained attention due to the ability to control film stiffness, making them highly adaptable for various applications.^82^ Qi et al. investigated the incorporation of functionalized GO layers into PSS/PAH polyelectrolyte multilayers (PEMs) using an LbL assembly via Langmuir–Blodgett (LB) deposition.^83^ Their study demonstrated a significant enhancement in the mechanical properties of the resulting multilayers. With just 8% GO content, the films achieved a Young’s modulus of 20 GPa.^83^ Such increases in stiffness provide a platform to probe how enhanced interfacial rigidity influences cytoskeletal tension, adhesion maturation, and force transmission at the cell–material interface.^84^ Similarly, Olek et al. utilized LbL assembly of polyethyleneimine (PEI) and carbon nanotubes (CNTs) to fabricate composites with an ultimate tensile strength of 150 ± 35 MPa and a Young’s modulus of 4.5 ± 0.8 GPa.^85^

Fabricating films with different thicknesses over rigid substrates provides a convenient method to modulate effective stiffness. In Figure 4A–C, Castilla-Casadiego et al. found that COL/HEP multilayers’ roughness increased with the number of bilayers; these variations were attributed to the inherent physical interactions of the polymers.^90^ They reported that increasing the number of bilayers gradually increased the thickness of the substrate, thereby modulating the Young’s modulus and overall mechanical properties of the multilayer films (Figure 4D), which in turn promoted stromal cell responses to the multilayered substrates.^74^ They studied how a layered top surface, either collagen or heparin, with and without interferon gamma (IFN-γ), can control both cell growth and immune activity in human mesenchymal stem cells (hMSCs). The results showed that IFN-γ typically slows cell growth, but when the surface was coated with heparin, the cells continued to grow and produced more immune-related signals.^98^ Pasquale et al. reported a monotonic decrease in the effective stiffness of diallyl dimethyl ammonium chloride/polystyrene sulfonate (PDDAC/PSS) films as the film thickness increased (10–50 bilayers).^99^ This inverse relationship demonstrates that cytoskeletal responses are sensitive not only to material composition but also to geometric factors such as film thickness, which alter how forces are transmitted at the interface.^99^ In addition, the elastic modulus of poly(L-lysine)/hyaluronic acid (PLL/HA) films^86^ was found to increase after capping with polystyrene sulfonate/poly(allylamine hydrochloride) (PSS/PAH) layers,^66^ where the penetration of PSS or PAH into the PLL/HA film was identified as a key factor contributing to stiffness enhancement.^99^ Chang et al. demonstrated that the surface Young’s modulus of PrS/DNA films could be tuned from 40 MPa to 86 kPa by adjusting the layer number from 4 to 24 bilayers that indicates a remarkable increase in selective endothelial cell adhesion efficiency with the increase in bilayer number.^100^ Haseli et al. investigated the surface stiffness influenced by *in situ* assembly of HEP/COL multilayers using QCM-D and observed a linear increase in film thickness with sequential deposition.^101^

### 3.2. Noncovalent Interactions

Noncovalent interactions in LbL films provide a reversible means of tuning film mechanics, allowing stiffness and viscoelasticity to be dynamically modulated through environmental parameters. The mechanical properties of LbL films are predominantly governed by the strength of polyelectrolyte interactions.^94^ Consequently, assembly parameters such as pH and ionic strength modulate the interactions between polyanions and polycations, especially in the case of weak polyelectrolytes that influence the extent of film swelling and thus the stiffness of the resulting LbL structures.^102^ The elastic modulus of PAH/PAA films varies between 200 kPa and 142 MPa, depending on the assembly pH, with values corresponding to pH 2.0, 4.0, and 6.5, respectively.^103^ For weak polyelectrolyte LbL films, variations in elastic modulus can be attributed to differences in ionic crosslinking between PAH and PAA chains under various pH conditions, with changes in swelling ratios also contributing to the observed differences.^103^ In another study, Chen et al. fabricated two-dimensional LbL gradient films by controlling the deposition pH of PAA (from 3 to 6.5) and PAH (from 11 to 7).^103^ The assembly processes were conducted in solutions with varying pH, which affected the polyelectrolyte conformation in the films and produced gradient films with varying thicknesses and an elastic modulus ranging from 500 to 2500 kPa.^103^

In addition, altering the salt concentration during film assembly can also affect the film rigidity.^104^ Figure 5 illustrates the use of quartz crystal microbalance with dissipation monitoring (QCM-D) to track multilayer assembly and mechanical changes during deposition.^105^ Gong and Gao investigated the effect of NaCl and NaBr, along with their concentrations, as noncovalent interactions on the assembly and compression behavior of polyelectrolyte multilayers.^105^ Using poly(diallyl dimethylammonium chloride) (PDADMAC) and poly(4-styrene-sulfonic acid-co-maleic acid, 1:1 SS:MA) sodium salt (PSSMA 1:1) as building blocks, they demonstrated that both the type and concentration of salts significantly influenced the compression ratio of the multilayers.^105^ Specifically, an increase in NaCl concentration, while maintaining a constant NaBr level, led to a higher compression ratio, indicating a progressive softening of the multilayers.^105^ Guzman et al. studied the stiffness of PSS/PDDAC films in relation to salt concentration, showing that altering the NaCl concentration (from 50 mM to 500 mM) changed the mechanical properties of the films.^106^

The stiffness of LbL films can also be influenced by humidity-driven swelling and softening.^107^ In the study of PAH/PAA polyelectrolyte multilayers (PEMs), a correlation between Young’s modulus and relative humidity was observed, with the plane strain Young’s modulus varying from 1 to 8 GPa as the relative humidity decreased from 90% to 10%.^108^ Additionally, Mameka et al. reported monitoring changes in the macroscopic length and elastic modulus of a nanoporous gold/polypyrrole (NPG/PPy) hybrid during the doping and de-doping process of the polymer in an aqueous electrolyte.^109^ Together, noncovalent interactions endow LbL films with a mechanically adaptive behavior, enabling dynamic tuning of stiffness and viscoelasticity to probe how cytoskeletal organization, adhesion dynamics, and force generation respond to changing interfacial mechanical cues.

### 3.3. Chemical Crosslinking

Chemical crosslinking improves the mechanical robustness of LbL films by making covalent bonds between functional groups on adjacent polymer chains, thereby reducing chain mobility and increasing network connectivity.^110^ In the context of mechanobiology, chemical crosslinking provides a means to stabilize interfacial stiffness, enabling investigation of how sustained mechanical cues influence cytoskeletal organization, adhesion maturation, and force transmission.^111^ These crosslinking mechanisms (Figure 6) have been shown to increase stiffness, stability, and cell–material interactions in a variety of polyelectrolyte and protein-based multilayer systems.^112^ Croll et al. investigated the effects of LbL deposition on a PEI-coated, decanethiol-modified QCM sensor in the absence and presence of EDC and NHS crosslinkers, monitoring the frequency and dissipation shifts at the third overtone.^113^ The film initially exhibited regular buildup; however, it was unstable upon the addition of subsequent layers.^113^

They mentioned that it was due to the instability in the formation of soluble macromolecular complexes between the previously deposited layer and the incoming polymer solution.^113^ After the addition of phosphate-buffered saline (pH 7.4), most of the films were rapidly lost.^113^ In contrast, when crosslinking agents were present, the frequency and dissipation shift for each layer were substantially higher, indicating the formation of thicker and more viscoelastic layers.^113^ Common crosslinking strategies include aldehyde-based agents, carbodiimide chemistry (e.g., EDC/NHS), and naturally derived crosslinkers such as genipin, which enable covalent stabilization of multilayer structures. Examples of crosslinking agents include dialdehydes,^115^ carbonamides (EDC/NHS),^116^ epichlorohydrin,^117^ genipin,^118^ and polyelectrolyte combinations, such as poly(L-lysine)/hyaluronic acid (PLL/HA),^86^ poly(L-lysine)/poly(glutamic acid) (PLL/PGA),^114^ chondroitin sulfate A/poly-L-Lysine (CSA/PLL),^119^ and CHI/HA^120^ during multilayer formation. Overall, chemical crosslinking introduces stable and tunable interfacial mechanical properties in LbL films, enabling systematic investigation of how persistent mechanical cues regulate cytoskeletal organization, adhesion maturation, and force transmission. In contrast to noncovalent interactions, which allow dynamic modulation, covalent crosslinking provides a platform to probe cytoskeletal behavior under mechanically stable conditions, offering complementary insights into mechanoregulation.

## 4. MECHANISTIC INSIGHTS INTO CYTOSKELETAL ORGANIZATION REVEALED BY MECHANICALLY TUNABLE INTERFACES

### 4.1. Tunable Mechanics Uncouple Protrusion, Contractility, and Adhesion

Mechanically tunable interfaces have demonstrated that protrusion, contractility, and adhesion, often considered inseparable aspects of cytoskeletal behavior, are instead regulated by distinct mechanical cues.^121^ On rigid glass or uniformly elastic gels, high effective stiffness simultaneously promotes actin polymerization, focal adhesion formation, and actomyosin contractility, making it difficult to determine cause-and-effect relationships.^122^ In contrast, mechanically tunable hydrogel and multilayer platforms decouple substrate stiffness from surface ligand chemistry, enabling independent control of mechanical and biochemical cues, revealing regulatory mechanisms that are invisible on static substrates.^58^ For example, bulk-tunable hydrogels primarily reveal stiffness-dependent transitions in contractility, whereas interfacial systems such as LbL films isolate adhesion-mediated force transmission by independently tuning surface mechanics and chemistry.^21^

Using such tunable systems, studies demonstrated that actin-driven protrusion and actomyosin contractility respond differently to mechanical resistance.^123^ Cells cultured on soft polyacrylamide hydrogels preferentially favor lamellipodia formation and dynamic actin polymerization, allowing exploratory protrusive behavior with limited force transmission.^123^ In contrast, increasing substrate stiffness specifically stabilizes stress fibers and boosts myosin II-dependent contractility, even when overall cell spreading remains similar.^121^ This finding shows that protrusive and contractile actin networks are not merely different force regimes of a single cytoskeletal state but are controlled by separate mechanosensitive pathways.

Mechanically tunable surfaces further clarify how non-muscle myosin II (NM II) activity integrates cytoskeletal tension with adhesion maturation.^124^ Increasing mechanical resistance elevates regulatory light chain phosphorylation, encouraging NM II to assemble into bipolar filaments and produce retrograde actin flow.^124^ This flow limits excessive protrusion while also strengthening nascent adhesions, helping them mature into load-bearing focal adhesions.^124^ Such force-dependent adhesion stabilization is difficult to resolve on rigid glass substrates, where adhesion growth and contractility are already maximized.^122^ The importance of the LbL assembled interface became clear when surface chemistry and charge were varied separately from bulk stiffness. In heparin-collagen multilayer systems, positively charged or adhesive multilayers improve integrin engagement and force transmission by the actomyosin, resulting in stronger stress fiber development and reinforcement of focal adhesions.^125,126^ Conversely, less adhesive or negatively charged films inhibit contractile coupling despite having similar bulk mechanical properties.^125^ These studies showed that force anchoring at the interface, not stiffness alone, determines whether actomyosin-generated tension is effectively transmitted to the substrate.

Engler et al. demonstrated that increasing substrate stiffness promotes focal adhesion growth and elongation, as visualized by paxillin immunofluorescence (Figure 7).^122^ In these experiments, stiffer substrates also increased the expression and recruitment of key focal adhesion and cytoskeletal proteins, including non-muscle α-actinin, filamin, talin, and focal adhesion kinase (FAK/PTK2), indicating coordinated reinforcement of adhesion and contractile machinery.^122^ Importantly, thin, soft polyacrylamide gels supported on glass allowed cell spreading like that on stiffer substrates yet exhibited significantly different cytoskeletal organization.^122^ Actin assembly closely followed adhesion formation, demonstrating that matrix stiffness, not cell area alone, drives actomyosin organization in mesenchymal stem cells.^122^ These fundamental findings established a stiffness-dependent link between adhesion growth and cytoskeletal tension but did not enable independent control of protrusion, contractility, and adhesion. Mechanically tunable interfaces extend this framework by allowing selective engagement of cytoskeletal modules rather than a single uniform response. In this broader mechanical context, recent studies demonstrated that crosslinking stiff microtubules to more compliant actin networks alters the effective mechanical behavior of the cytoskeleton.^127^ These findings highlight that hybrid filament architectures can modulate force transmission and cytoskeletal mechanics in ways that cannot be predicted by substrate stiffness alone.

These findings show that mechanically tunable interfaces act as selective mechanical filters, allowing the experimental separation of protrusion, contractility, and adhesion. This insight fundamentally changed our understanding of cytoskeletal regulation by demonstrating that mechanical inputs do not influence the cytoskeleton globally but instead target specific modules based on how forces are resisted and transmitted at the interface.

### 4.2. Mechanical Coupling Drives Alignment, Polarity, and Collective Cytoskeletal States

While the preceding sections focus on cell-based systems, complementary insights into cytoskeletal organization have emerged from reconstituted and minimal systems, where specific mechanical interactions can be isolated and studied in the absence of complex cellular regulation. These reductionist approaches have demonstrated that cytoskeletal organization is not determined solely by local molecular interactions but can emerge from long-range mechanical coupling and force redistribution.^128,129^ When forces generated by molecular motors and polymerizing filaments are transmitted through compliant, anisotropic, or mechanically structured environments, cytoskeletal systems develop collective behaviors, including alignment, polarity, and active flows, which are not observed on rigid glass or isotropic elastic substrates.^128,129^

This principle is evident in reconstituted active matter systems, where mechanically coupled interfaces enable the emergence of collective cytoskeletal order.^128^ In microtubule-kinesin assemblies formed on mechanically permissive surfaces, motor-driven filament sliding produces long-range nematic alignment and sustained active flows that depend on surface-mediated force transmission and boundary conditions.^128^ These findings demonstrate that collective cytoskeletal organization can arise from mechanical coupling alone, even in the absence of higher-order biochemical signaling.^128^ Similar principles emerge at the cellular scale, where mechanically anisotropic and micropatterned substrates impose directional constraints on force transmission.^129,130^ For example, microchanneled interfaces drive robust F-actin alignment parallel to the channel axis and enhance contractile output even under spatially uniform biochemical conditions, indicating that cytoskeletal orientation can be mechanically imposed rather than biochemically specified.^129^

This concept is illustrated directly by Yim et al., who showed that human mesenchymal stem cells (hMSCs) cultured on unpatterned surfaces display a dense, isotropic F-actin network, whereas cells grown on nanogratings adopt elongated morphologies with parallel, lower-density stress fibers^130^ shown in Figure 8. Notably, similar cytoskeletal organization was observed on both tissue-culture polystyrene (TCPS) and PDMS substrates, leading the authors to conclude that nanotopography exerts a stronger influence on F-actin organization than bulk substrate stiffness.^130^ These results demonstrate that mechanically imposed anisotropy can dominate cytoskeletal architecture independently of elastic modulus.

Immunofluorescence staining of phospho-Y397 focal adhesion kinase (pFAK) and vinculin revealed well-defined focal adhesions on unpatterned controls, with pFAK localized both centrally and peripherally at the termini of F-actin bundles within filopodia and lamellipodia.^130^ Surfaces functionalized with adhesive proteins or peptides enhance integrin engagement, promoting the assembly of focal adhesions coupled to actin stress fibers.^125^ *In vitro*, these structures anchor the cytoskeleton and support directed migration, while in living tissues, focal adhesion-like complexes remain smaller and more transient yet still guide collective cell movement through dynamic force transmission.^131^

Surface chemistry further modulates these mechanically driven behaviors. Hydrophilic, positively charged multilayers promote robust cell attachment and well-organized actin cytoskeletal structures, whereas more hydrophobic or negatively charged films hinder adhesion and disrupt force transmission.^125^ Consistent with this, Kandel et al. demonstrated that heparin–collagen (HEP–COL) layer-by-layer assemblies influence actomyosin contractility and intracellular force generation, providing a mechanically permissive microenvironment for probing cytoskeletal remodeling and mechanotransduction.^126^

Mechanical coupling also extends to microtubule organization and the establishment of polarity.^132^ Several studies reported that microtubule stabilization induced by heparin–collagen matrices facilitates the formation of front–back polarity, resulting in sustained directional migration and enhanced persistence.^132,133^ These findings demonstrate that mechanically structured environments reinforce feedback between actin and microtubule systems, enabling polarized cytoskeletal states to emerge through force-mediated crosstalk.^134–136^

Related force-feedback mechanisms were observed in reconstituted branched actin networks, where mechanical loading increases filament density and network stiffness, thereby enhancing myosin motor performance and reinforcing collective alignment.^137^ Together, these results establish that cytoskeletal systems can self-organize into aligned, polarized, and collective states through mechanical feedback loops rather than solely through molecular regulation. Adhesive ligands and peptides enhance integrin engagement, promoting the assembly of focal adhesions coupled to actin stress fibers.^125^ *In vitro*, these structures anchor the cytoskeleton and support-directed migration, while in living tissues, focal adhesion-like complexes remain smaller and more transient yet still guide collective cell movement through dynamic force transmission.^131^

### 4.3. Cytoskeletal Mechanosensing Across Scales

Mechanically tunable interfaces have transformed the understanding of cytoskeletal mechanosensing by revealing that cellular responses to mechanical cues are highly scale-dependent and governed by thresholds, optima, and feedback mechanisms rather than simple linear responses to substrate rigidity.^122,138^ Early substrate stiffness studies established that cells respond to the mechanical properties of their environment, but mechanically tunable hydrogels, multilayer films, and three-dimensional matrices enabled deeper investigation into how forces are transmitted across adhesion structures, cytoskeletal networks, and the nucleus.^139^ Bulk-tunable systems have been particularly important for identifying stiffness-dependent transitions in spreading, contractility, and differentiation, whereas interfacial platforms provide insight into how local surface mechanics regulate force transmission and mechanotransductive signaling at the cell–material interface.^3^

One important insight gained from mechanically tunable systems is the connection between cytoskeletal tension and nuclear deformation.^140^ Actomyosin-generated forces transmitted through adhesion sites and perinuclear actin structures have been shown to influence nuclear shape and mechanics, directly linking extracellular mechanical resistance to nuclear architecture and gene expression.^140^ This relationship cannot be observed on rigid substrates, where nuclear deformation is mostly saturated and unresponsive to further mechanical changes. The importance of force-bearing cytoskeletal architecture becomes even more apparent in three-dimensional mechanically tunable matrices.^139^ In confined 3D environments, cells assemble distinct actin structures, including perinuclear actin caps and contractile networks that span from adhesions to the nucleus, enabling force transmission across the entire cell.^139^ These architectures are essential for migration through confined spaces and highlight that cytoskeletal mechanosensing is inherently geometry- and dimensionality-dependent.

Foundational studies by Engler and Georges laid the conceptual groundwork for stiffness-dependent regulation of the cytoskeleton by showing that substrate elasticity affects cell shape, adhesion formation, and actomyosin structure.^122^ However, interfaces that can be mechanically tuned expanded on these findings by revealing responses that are not simply linear.^2,141,142^ On very soft substrates, actomyosin networks stay diffused and weakly organized, while overly stiff substrates lead to large focal adhesions and isometric contraction.^2,141^ In contrast, stiffness regimes near that of relaxed muscle provide optimal mechanical conditions for actomyosin striation, effective force propagation, and coordinated cytoskeletal architecture.^141,143^ These findings support the idea that mechanosensing depends on specific stiffness optima rather than a straightforward linear relationship. These nonmonotonic, stiffness-dependent regimes of cytoskeletal organization are schematically summarized in Figure 9, which illustrates the transition from diffuse actomyosin networks on soft substrates to optimal striation at intermediate stiffness and rigid, isometrically contracted states on very stiff matrices. Beyond bulk and interfacial mechanics, cytoskeletal mechanosensing is further influenced by the intrinsic mechanical properties of filament networks.^144^ Recent studies demonstrated that crosslinking stiff microtubules to more compliant actin networks changes the effective mechanical response of the cytoskeleton, reshaping force distribution and resistance.^127^ These hybrid architectures show that cytoskeletal stiffness is not an intrinsic material property, but an emergent feature determined by filament coupling and network connectivity.^127^

Taken together, these findings illustrate that different classes of mechanically tunable platforms provide access to distinct aspects of cytoskeletal regulation. Bulk systems reveal stiffness-dependent thresholds and optima, micropatterned and anisotropic substrates uncover geometry- and force-guided organization, and interfacial platforms enable precise control of adhesion-mediated force transmission. Integrating these approaches provides a more comprehensive understanding of how mechanical inputs regulate cytoskeletal organization across scales.

## 5. OUTLOOK OF CYTOSKELETAL STUDIES ON MECHANICALLY TUNABLE SURFACES

Future studies in cytoskeletal mechanobiology will likely move beyond simplified stiffness-response models toward understanding how distinct mechanical cues regulate specific cytoskeletal processes, including protrusion, adhesion, contractility, and force transmission across multiple biological scales. Whereas early work established that cells respond to substrate rigidity, many open questions now center on how mechanical information propagates from molecular motors and filament assemblies to whole-cell behaviors and tissue-level organization.^145^ Developing evidence suggests that cytoskeletal mechanosensing is highly dynamic, nonlinear, and strongly influenced by geometry, confinement, and mechanical history.^146^ Mechanically tunable systems are therefore evolving from platforms used primarily to perturb stiffness into experimental frameworks for probing the physical principles governing cytoskeletal organization and force generation.

An important direction for the field will be the integration of cell-based and reductionist approaches. Cell-based systems remain essential for understanding how mechanosensitive pathways regulate migration, differentiation, and force transmission in physiologically relevant environments, but the complexity of cellular signaling often obscures the underlying mechanical mechanisms.^147^ In parallel, reconstituted and minimal cytoskeletal systems are increasingly being used to isolate the intrinsic mechanical behaviors of filament–motor assemblies under controlled conditions.^148^ Combining these approaches may help distinguish which emergent behaviors arise directly from physical interactions within cytoskeletal networks and which require higher-order regulation, providing a path toward identifying the physical rules that govern cytoskeletal organization. Mechanically tunable interfaces are particularly valuable in this context because they enable comparable mechanical conditions to be examined across molecular, network, and cellular scales.^149^

Future mechanically tunable systems will also likely incorporate increasingly complex and dynamic mechanical environments that more closely resemble physiological conditions. Rather than relying solely on static elastic substrates, emerging platforms integrate viscoelasticity, anisotropy, stress relaxation, spatial heterogeneity, and time-dependent mechanical changes. Hybrid systems that combine tunable mechanics with controlled ligand presentation, confinement, or force sensing may provide new opportunities to investigate how multiple mechanical cues are integrated simultaneously by cytoskeletal systems.^150^ Such platforms may also enable independent control of bulk and interfacial mechanics, providing new opportunities to determine how distinct mechanical cues selectively engage protrusive, adhesive, and contractile cytoskeletal modules. These approaches may be especially important for understanding processes such as collective migration, active force redistribution, and mechanically driven cytoskeletal remodeling in three-dimensional environments.^151^

Another major challenge moving forward will be connecting force generation and force transmission across scales through quantitative and predictive frameworks. Coupling mechanically tunable systems with high-resolution imaging, traction force measurements, microrheology, optical tweezers, and computational modeling will be important for linking molecular-scale force generation with emergent cytoskeletal organization and cell behavior. More broadly, integration of mechanically tunable systems with theoretical approaches from soft matter physics and active matter theory may help establish predictive models for how cytoskeletal systems organize under defined mechanical constraints.

Ultimately, no single mechanically tunable platform can fully capture the complexity of cytoskeletal mechanobiology because force transmission and mechanosensing are inherently multi-scale processes. Continued progress will therefore depend on integrating complementary experimental systems that bridge bulk mechanics, interfacial mechanics, and reductionist reconstituted approaches. As mechanically tunable approaches become increasingly quantitative and physiologically relevant, they will not only advance fundamental understanding of cytoskeletal organization but also inform the development of engineered tissues, mechanotherapeutic strategies, and adaptive biomaterials that actively respond to mechanical cues.

## Figures and Tables

**Figure 1.**
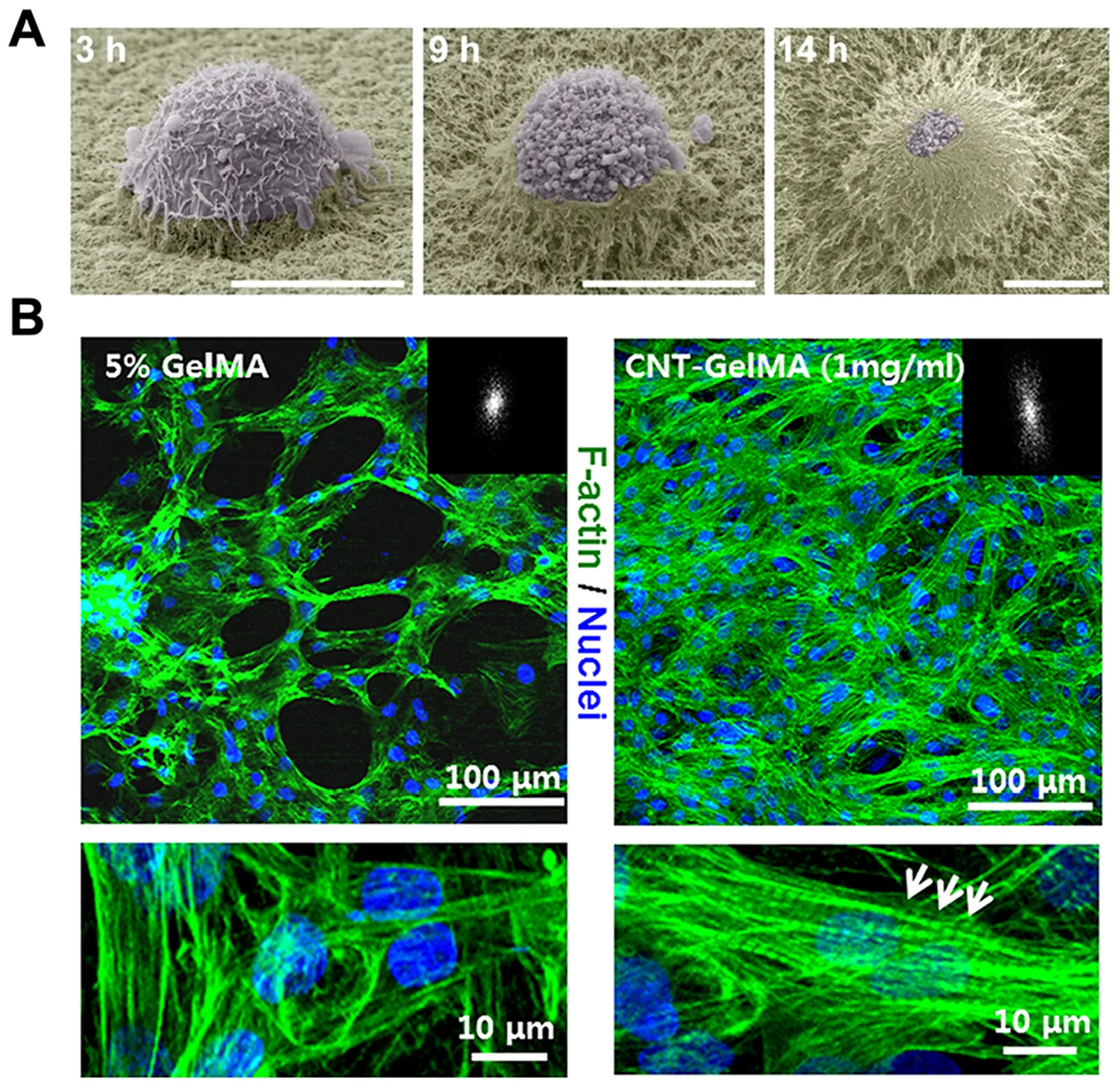
Uses of Matrigel and hydrogel for studying cytoskeletal dynamics. (A) Scanning electron micrographs of MDA-MB-231 cells invading a thick layer of Matrigel at the indicated times after plating. (Scale bars, 10 μm). Reproduced from ref 19. Available under a CC-BY 4.0 license. Copyright 2021, Poincloux et al. (B) Confocal images of cardiomyocytes cultured for 5 days on pristine GelMA and 1 mg/mL CNT–GelMA show more uniform cell distribution and partial alignment on CNT–GelMA. F-actin and nuclei were labeled in green and blue, respectively. Insets show the corresponding FFT images. Higher-magnification images reveal well-elongated cardiac cells with distinct F-actin cross-striations on CNT–GelMA (bottom right, white arrows), which were not observed on pristine GelMA (bottom left). Reproduced from ref 20. Copyright 2013, American Chemical Society.

**Figure 2.**
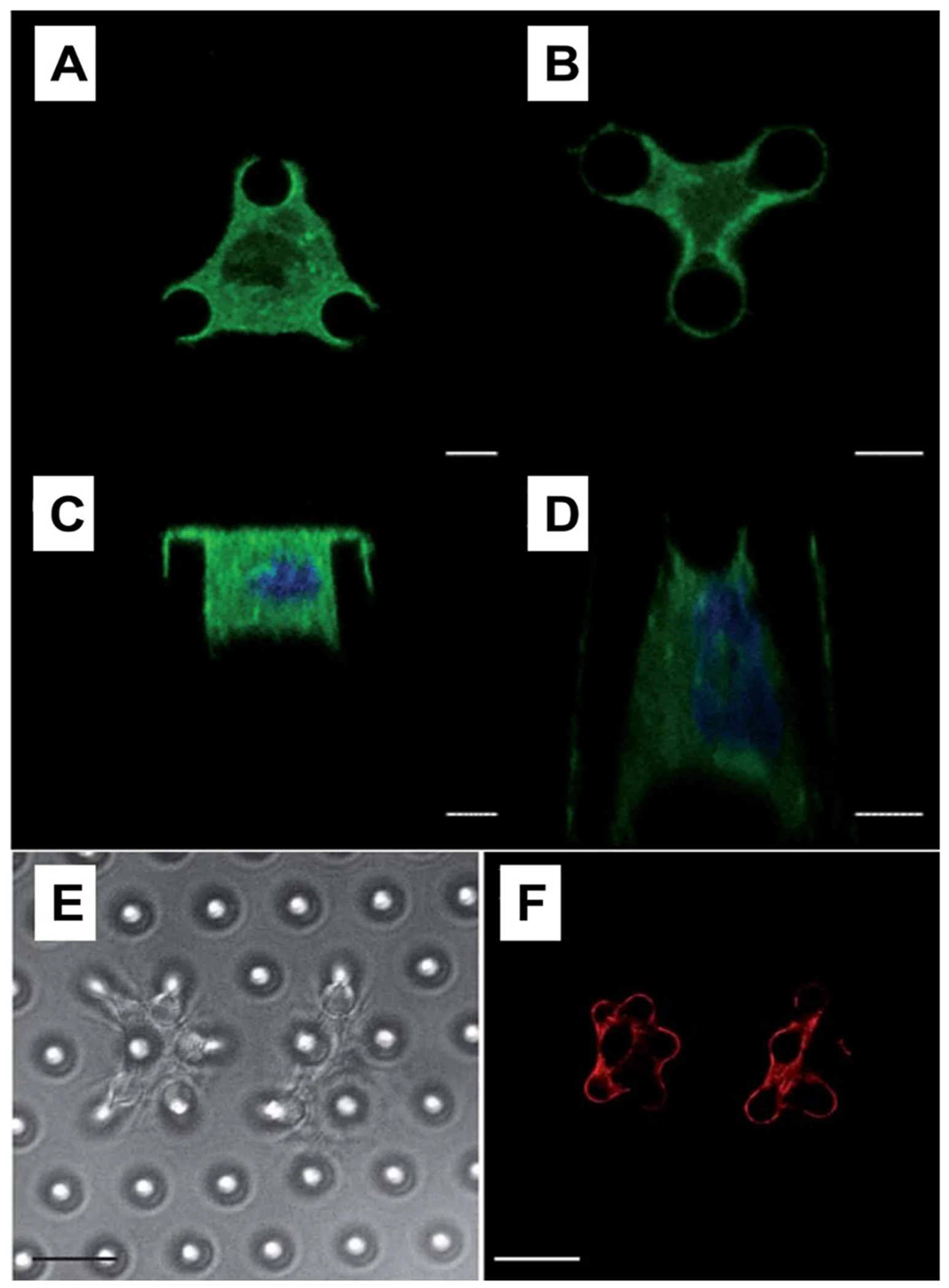
Spreading of REF52 cells into PDMS micropillars. Living cells were first labeled with calcein (green), then fixed for nucleus staining with DAPI (blue). Image stacks were taken with a confocal microscope. (A) Cells were fixed 15 min after their adhesion on the substrate. (B) Cells were fixed 60 min after their adhesion on the substrate. Top views are taken at a height of 5 μm above substrate (A, B) and their corresponding side views are shown below (C, D) (Scale bar, 5 μm). (E) Bright-field image of micropillar deflection caused by spreading of two individual cells (scale bar, 15 μm). (F) Cell morphology after spreading on micropillar substrates, shown by immunofluorescent staining of actin. Cells were fixed 90 min after plating (scale bar, 15 mm). Reproduced with permission from ref 46. Copyright 2011, Royal Society of Chemistry.

**Figure 3.**
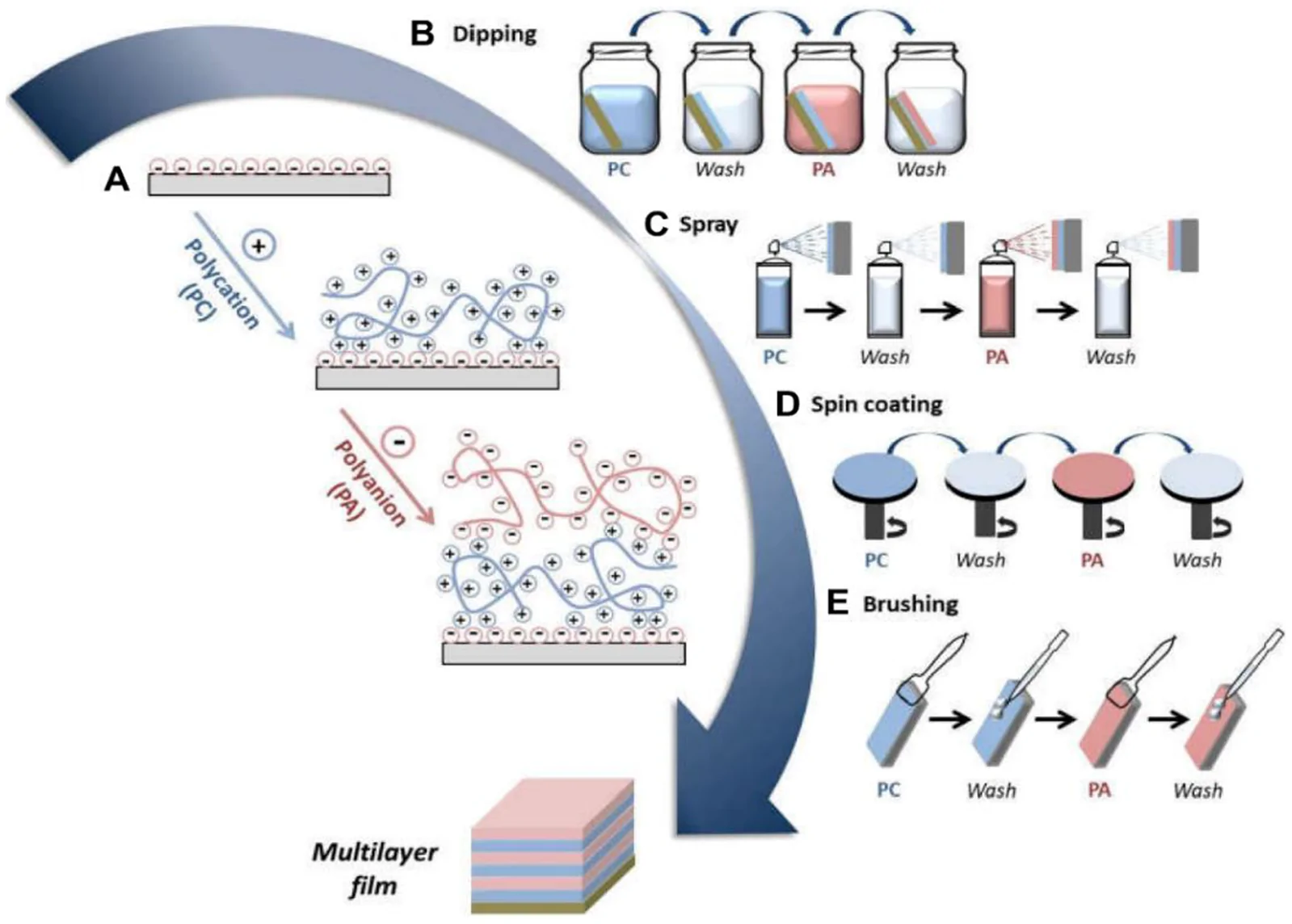
Strategies for LbL film assembly. (A) Schematic illustration of the sequential deposition of polycations (PCs) and polyanions (PAs) during LbL assembly, resulting in the formation of a multilayer film. Various LbL deposition techniques include sequential (B) dipping, (C) spraying, (D) spin coating, and (E) brushing. Reproduced from ref 58. Available under a CC-BY 4.0 license. Copyright 2021, Criado-Gonzalez et al.

**Figure 4.**
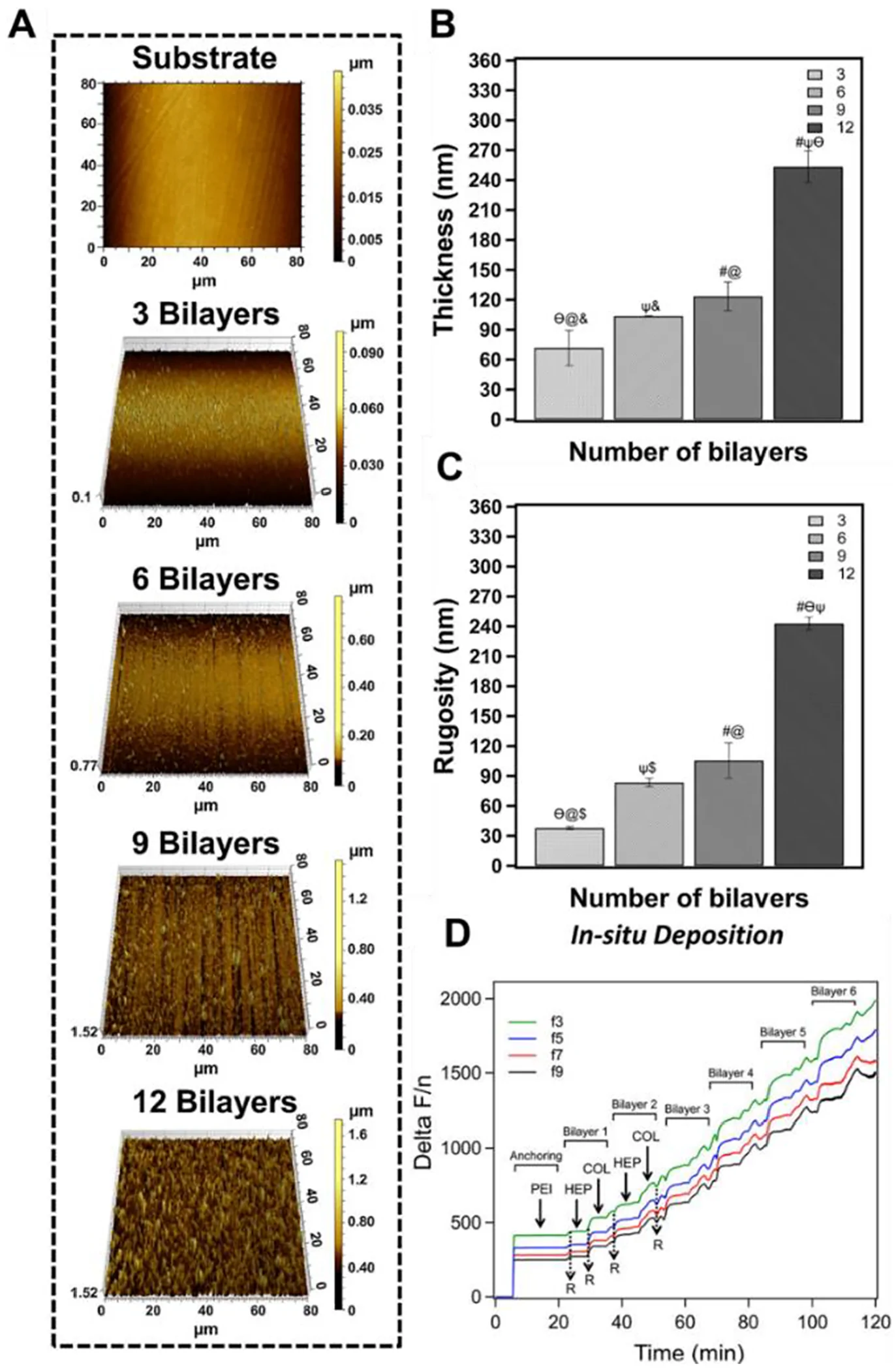
Surface morphology and stiffness changes during HEP/COL multilayer formation. (A) AFM-measured surface morphology of the substrate and films with 3rd, 6th, 9th, and 12th bilayers. (B) Film thickness as a function of bilayer number. (C) Surface roughness as a function of bilayer number. Error bars represent standard deviation. Statistical significance: *p* < 0.01 is indicated by #, @, &, ψ, and θ *p* < 0.05 is indicated by $, %, and ! Bars containing the same symbol are statistically different. Reproduced with permission from ref 90. Copyright 2018, Elsevier. (D) *In situ* deposition monitored by QCM-D (R indicates the rinse step). Error bars represent the standard deviation (*n* = 3). Statistical significance is denoted as follows: *p* < 0.01 is indicated by #, @, and &, and *p* < 0.05 is indicated by $ and %. Bars sharing the same symbol are statistically different from each other. Reproduced from ref 74. Copyright 2020, American Chemical Society.

**Figure 5.**
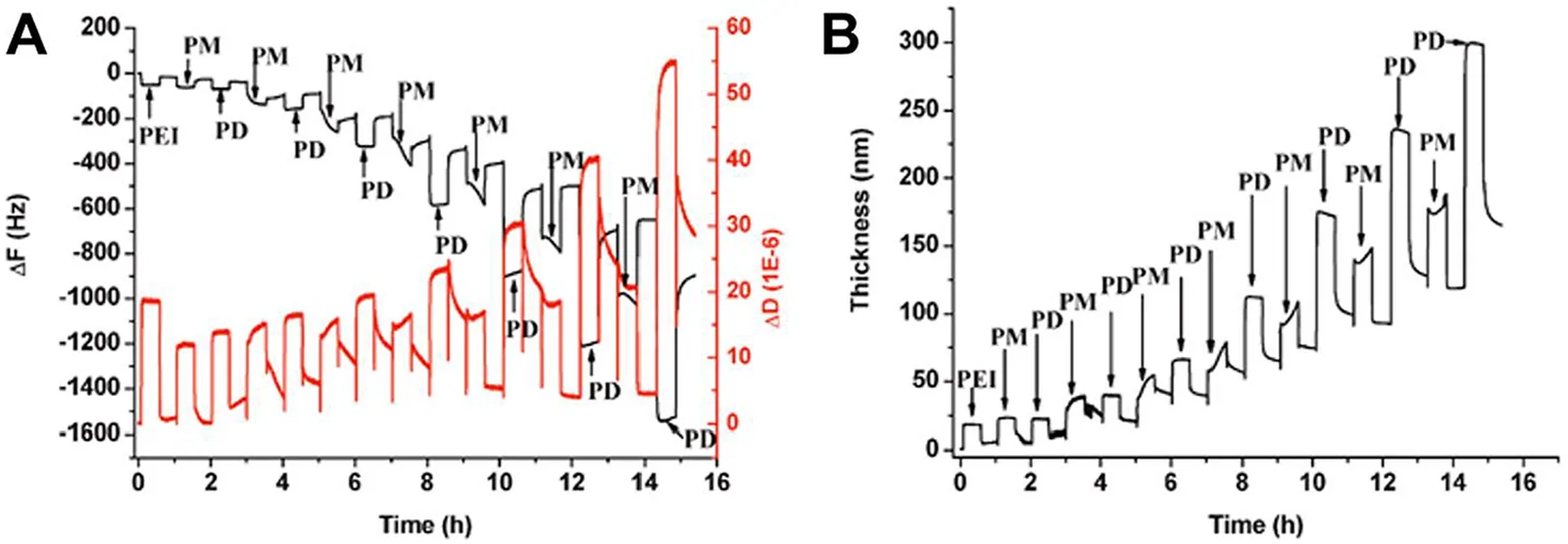
Real-time QCM-D analysis of PSSMA/PDADMAC multilayer formation. (A) Changes in frequency (Δ*F*) and dissipation (Δ*D*) during formation of PSSMA/PDADMAC multilayers in 1 M NaBr solution, where the overtone number *n* = 3. (B) Corresponding wet thickness as a function of the layer number. PM and PD represent PSSMA and PDADMAC in this figure, respectively. Reproduced with permission from ref 105. Copyright 2009, Royal Society of Chemistry.

**Figure 6.**
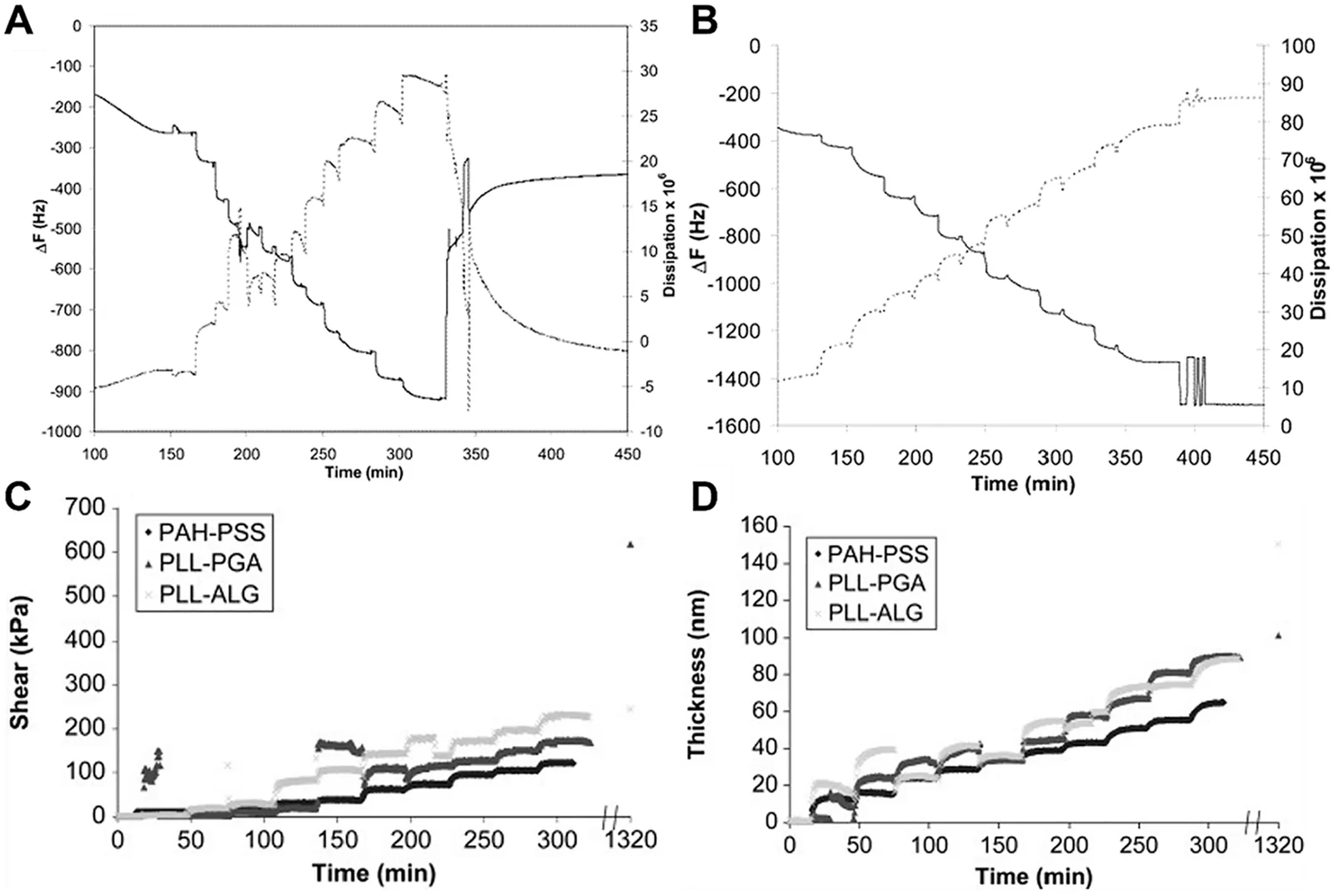
Effects of crosslinking agents on surface stiffness. (A) Frequency (solid line) and dissipation (dashed line) shifts during LbL assembly without a crosslinking agent. Instability appears after the first three layers. The buffer was changed to PBS at 330 min, after which most of the film was lost. (B) Frequency (solid line) and dissipation (dashed line) shifts during LbL assembly with EDC/NHS on a PEI-coated decanethiol substrate. Well-defined, stable layers form with no detectable instability. The film shows moderate swelling upon exposure to PBS at *t* = 390 min, but no significant mass loss occurs over repeated PBS/MES exchange cycles. Reproduced from ref 113. Copyright 2006, American Chemical Society. (C) Shear modulus, (D) thickness of PAH–PSS, PLL–ALG, and PLL–PGA films during LbL assembly, as measured by QCMD. Data points along the right vertical axis are taken after film exposure to chemical crosslinking agents. Reproduced with permission from ref 114. Copyright 2008, Elsevier.

**Figure 7.**
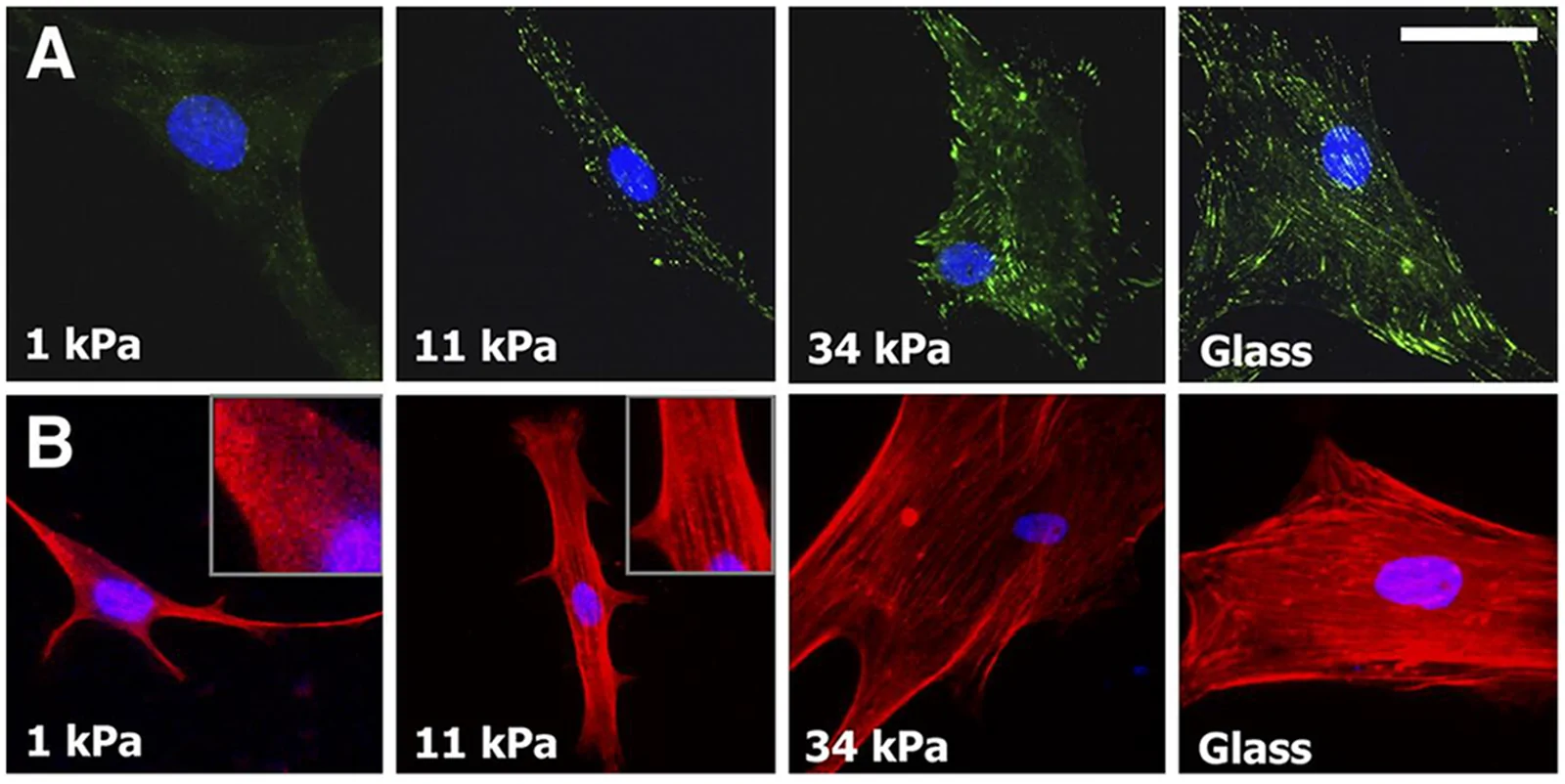
(A) Paxillin-labeled adhesions develop from undetectable, diffuse “contacts” on soft neurogenic gels (1 kPa) to distinct punctate adhesions on stiffer myogenic gels (11 kPa). On the stiffest osteogenic gels (34 kPa), adhesions appear long, thin, and slightly more peripheral than those on glass. (B) F-actin organization shows a similar pattern, shifting from diffuse on soft gels to well-formed stress fibers on stiffer substrates (Scale bar, 20 μm). Reproduced with permission from ref 122. Copyright 2006, Elsevier.

**Figure 8.**
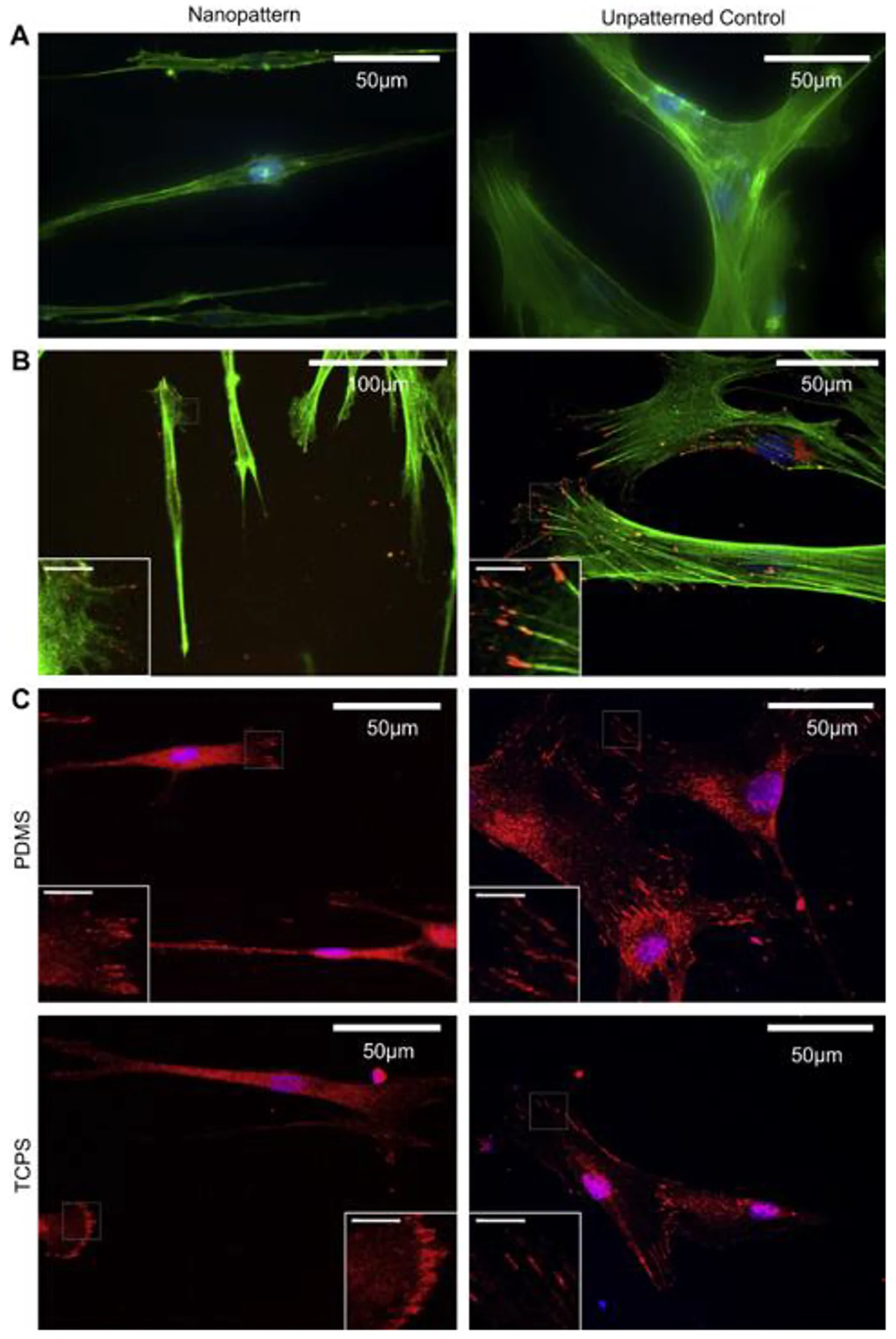
Nanotopography induces cytoskeletal alignment and focal adhesion reorganization in hMSCs. (A) F-actin cytoskeleton visualized with Oregon Green–labeled phalloidin in hMSCs cultured on PDMS substrates with 350 nm gratings or on unpatterned PDMS. (B) Distribution of focal adhesions shown by immunofluorescence staining of phospho–tyrosine-397 FAK (pFAK, red) along with F-actin (green). (C) Distribution of focal adhesions visualized by immunofluorescence staining of vinculin (red). Gray boxes indicate regions shown at higher magnification in the inset images (scale bar, 10 μm). Images in panel (A) were captured using fluorescence microscopy, while images in panels (B,C) were obtained with confocal microscopy. Reproduced with permission from ref 130. Copyright 2010, Elsevier.

**Figure 9.**
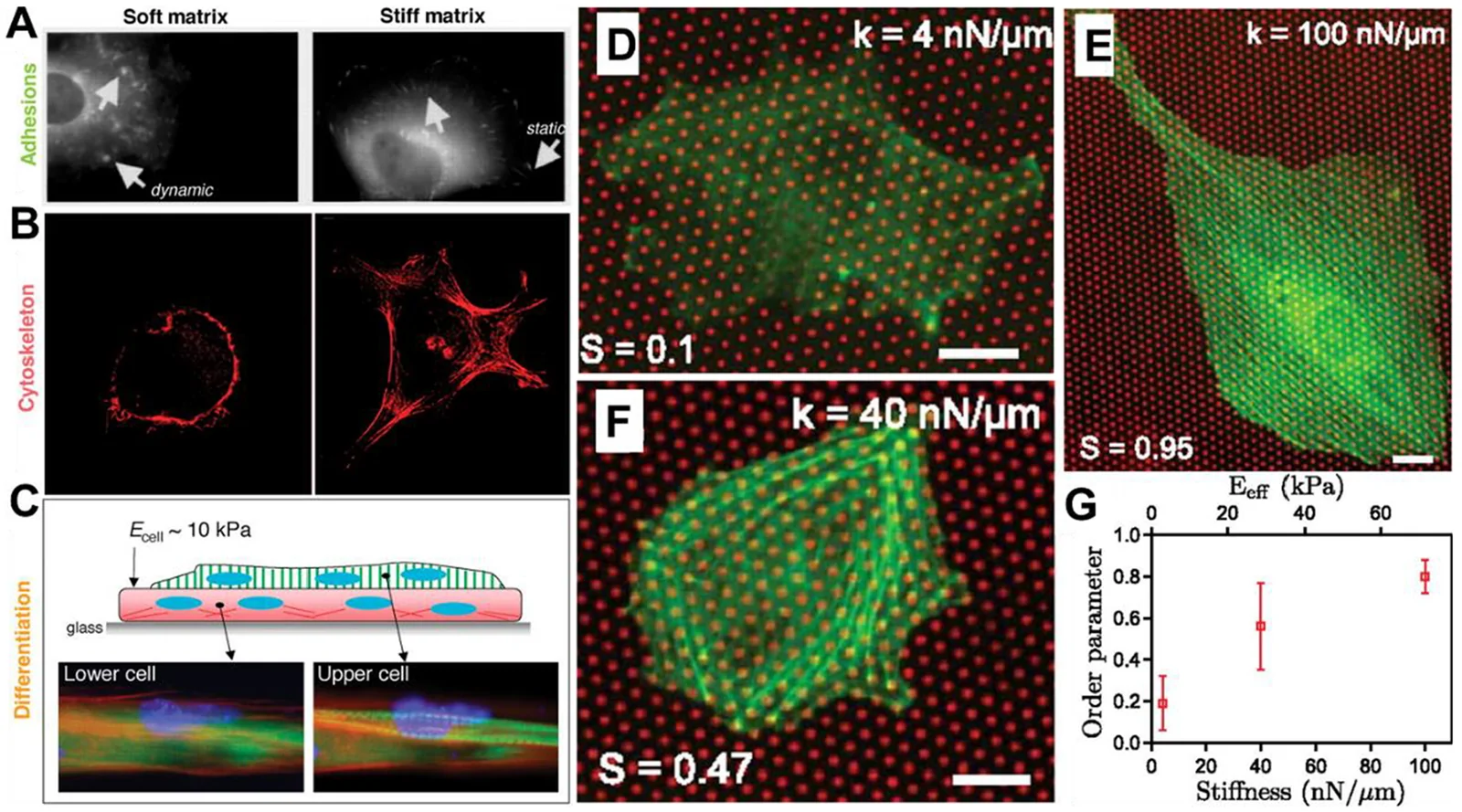
Effects of substrate stiffness on cell shape, cytoskeletal arrangement, and adhesion behavior. (A) Substrate stiffness influences adhesion and dynamics, cytoskeletal assembly, cell spreading, and differentiation outcomes such as myotube striation. Dynamic adhesions are found on soft substrates, while stable focal adhesions develop on stiff substrates. Reproduced with permission from ref 2. Copyright 1997, National Academy of Sciences. (B) Fluorescence images of fibroblasts stained with rhodamine–phalloidin to label F-actin show a lack of well-defined stress fibers in cells on soft gels (left). Conversely, fibroblasts on stiff gels (right) display prominent, organized stress fibers similar to those in cells on tissue culture plastic. Reproduced from ref 143. Available under a CC-BY 4.0 license. Copyright 2005, Georges et al. (C) Cell-on-cell culture system where a lower cell layer is first attached to glass, creating a mechanically flexible, cell-derived substrate sensed by an upper layer of myoblasts that then unite. Reproduced from ref 141. Available under a CC-BY 4.0 license. Copyright 2004 Engler et al. (D–G) Representative diagrams and actin staining depicting stress fiber organization on substrates of increasing stiffness: soft (~4 nN/μm) (D), intermediate (~40 nN/μm) (E), and stiff (~100 nN/μm) (F). Actin stress fibers (green) become more aligned with increased substrate stiffness. The scalar order parameter, *S*, measures fiber alignment, where θ indicates the angle of each stress fiber relative to a reference axis and θ_0_ its average orientation. *S* rises with stiffness (~0.1, 0.47, and 0.95, respectively). (Scale bar, 15 μm). (G) average order parameter (*S*) as a function of pillar spring constant and effective substrate rigidity (E _eff) for cells on substrates of three stiffness levels. Data were collected from ≥15 cells per condition. Reproduced from ref 142. Available under a CC-BY 4.0 license. Copyright 2012, Trichet et al.

**Table 1.** LbL Substrate Mechanical Properties^a^

polycation	polyanion	Young’s modulus	references
poly(L-lysine) (PLL)	hyaluronic acid (HA)	1–50 kPa	86
chitosan (CHI)	alginate (ALG)	10–500 kPa	87
poly(allylamine hydrochloride) (PAH)	poly(styrene sulfonate) (PSS)	100–1000 MPa	66
poly(ethylenimine) (PEI)	dextran sulfate (DS)	30–90 MPa	88
gelatin	alginate	135–331 kPa	89
poly(ethylenimine) (PEI)	poly (sodium-pstyrene sulfonate) (PSS)	15,000 kPa	90
collagen (COL)	heparin (HEP)	9000 kPa	90
chitosan (CHI)	hyaluronan (HA)	15 kPa	91
collagen	chondroitin sulfate	0.5–20 kPa	92
silk fibroin	heparin	10–300 kPa	93
poly(allylamine)	poly(styrene sulfonate) (PSS)	500–750 MPa	94
poly(vinyl alcohol) (PVA)	poly(acrylic acid) (PAA)	10–400 kPa	95
poly(diallyldimethylammonium) PDADMA	poly(styrene sulfonate) (PSS)	140 kPa	94
poly(ethylene glycol)-based polycations	DNA	1–50 kPa	96
poly(dopamine)	heparin	5–50 kPa	97

a Example materials used to assemble LbL films and their Young’s modulus.
